# Clinical outcomes of coronary artery bifurcation disease patients underwent Culotte two-stent technique: a single center experience

**DOI:** 10.1186/s12872-019-1192-2

**Published:** 2019-09-02

**Authors:** Chih-Feng Chang, Keng-Hao Chang, Chih-Hung Lai, Tzu-Hsiang Lin, Tsun-Jui Liu, Wen-Lieng Lee, Chieh-Shou Su

**Affiliations:** 1 Cardiovascular Center, Taichung Veterans General Hospital, 1650, Taiwan Boulevard Sect. 4, Taichung, 40705 Taiwan; 2 Department of Internal Medicine, Division of Cardiology, Taichung Armed Forces General Hospital, Taichung, Taiwan; 3 Department of Internal Medicine, Cheng Ching Hospital, Taichung, Taiwan; 4 Institute of Clinical Medicine, and Department of Medicine, National Yang-Ming University School of Medicine, Taipei, Taiwan; 5 Department of Surgery, National Yang Ming University School of Medicine, Taipei, Taiwan; 6 Department of Medicine, National Yang Ming University School of Medicine, Taipei, Taiwan

**Keywords:** Coronary artery bifurcation disease, Culotte two-stent technique, Clinical outcomes

## Abstract

**Background:**

Percutaneous coronary intervention for coronary artery bifurcation disease (CABD) remains challenging. In patients of CABD with situations that two-stent strategy is needed, the culotte technique is a widely used method and also as the majority at out institution. We sought to take a look of the clinical outcomes of our culotte stenting patients.

**Methods:**

This retrospective study analyzed 238 consecutive CABD patients who underwent culotte two-stent technique at a tertiary medical center between July 2008 and November 2015.

**Results:**

Culotte technique was used in 238 lesions in 238 patients. Of these patients, all DES were implanted for culotte two-stent technique. Most of these patients were elderly, male gender, ACS on admission and multiple vessel disease. The bifurcation lesions were mostly located at left coronary artery (51.3%), categorized as true bifurcation lesion (92%) and calculated less than 70 degree of bifurcation angle (74.4%). During a medium 3.27-year follow up, the angle of bifurcation lesion ≥70° and body mass index were positively independent predictors for target lesion failure (TLF), diabetes mellitus was an independent predictor of target vessel revascularization, and statin therapy for hyperlipidemia, hemoglobin and EF were negatively independent factors associated to total mortality. The rates of in-hospital and total mortalities were 4.2 and 17.6%.

**Conclusion:**

In this cohort of CABD patients with most left main and left anterior descending artery lesions treated by culotte stenting, the procedural success rate was high and the intermediate clinical outcomes were acceptable. *(Reviewer #1, Comment #1)* Bifurcation angle (≥ 70°) is an inherently independent predictor of TLF and other two-stent strategy replaced needed to be considered in this situation.

## Background

Coronary artery bifurcation disease (CABD) occurs in 15–20% of coronary artery disease (CAD) patients undergoing percutaneous coronary intervention (PCI) [1, 2] and remains a challenge in interventional cardiology even though coming modern interventional stent and technique in the last decades. To the best of our knowledge, there was no definite one strategy superior to other strategy in treating this kind of complex coronary lesion, but a provisional strategy has been accepted as the default technique to treat CABD by the majority of operators because the provisional strategy itself can reduce procedural biomarker release, and save procedure times, and radiation and contrast dose with similar major adverse cardiovascular events (MACE) at short-term clinical follow-up [3–8]. However, two-stent strategy was practically needed for this kind of complex coronary lesion in some specific situations (e.g. severe ostial disease of the side branch, dissection or diminished flow, a large territory at risk in the side branch etc.). Culotte two-stent strategy is the most applied technique used for CABD in our institute and we therefore undertook a look of the clinical outcomes of patients treated with the culotte two-stent technique at our institution.

## Methods

### Study population

A retrospective analysis was conducted from the all patients with CABD that underwent PCI utilizing culotte two-stent strategy in Taichung Veterans General Hospital from July 2008 to November 2015. The classification of CABD was sorted by Medina classification system and the true bifurcation disease was defined as Medina classification 1,1,1, 1,0,1, or 0,1,1. Exclusion criteria included a history of PCI for acute myocardial infarction (AMI) with cardiogenic shock, in-stent restenosis at coronary bifurcation, and bifurcations lesions treated with bare metal stent (BMS).

The standard technique of culotte stenting for CABD was employed and presented briefly as followings. After placement of the guiding catheter, 2 wires were inserted in the distal bed of the main and side branches. Balloon angioplasty was conducted by sequential inflation of a semi-compliant balloon in each branch using an equal size or down quarter size of reference vessel. After balloon angioplasty, the first stent was introduced in the branch which was a smaller branch, a more difficult branch to deliver originally or a difficult passing via stent strut after the first stent deployment, and deployed using regular pressure. Stent length was chosen in order to cover the proximal part before the bifurcation, and the distal part of the lesion in the branch. A third wire was used to recross the strut of the first stent and went into the main branch using the first side branch wire as a marker. Afterward, the first wire in the main was removed and a small balloon was used to open the struts and prepare the way for the second stent. In some cases, the operator would perform kissing balloon technique of main and side branches at this stage to reduce the retraction effect of stent opposite site when the stent strut was opened up. This was called first kissing balloon technique of culotte stenting. Afterward, a second stent of the same type as the first (but of different length if lesion length differed) was inserted crossing the strut of the first stent and placed in order to cover the proximal and distal parts of the main branch with overlapping of the first stent in the proximal part of the bifurcation. After deployment of this stent, a wire was rewired into the side branch via the second stent strut and the stent strut was opened up as previously described. At the end of culotte stenting, a simultaneous kissing balloon inflation was performed and usually indicated the second kissing balloon technique in the case that the first kissing balloon technique was done or final kissing balloon technique in the case which there was no first kissing balloon technique done. Drug-eluting stent (DES) for culotte stenting was conducted in all cases. DES used in this study included sirolimus-eluting stents [(Cypher family, Cordis Corporation, Johnson & Johnson, Warren, NJ,), (Ultimaster, Terumo Corporation, Tokyo, Japan), and (Orsiro, Biotronik AG, Buelach, Switzerland)], paclitaxel-eluting stents (Taxus family, Boston Scientific, Natick, MA), zotarolimus-eluting stent (Endeavor, Endeavor Resolute, and Endeavor Integrity, Medtronic, Santa Rosa, CA), everolimus-eluting stents [(Xience family, Abbott Vascular, Santa Clara, CA), and Promus family (Boston Scientific)], biolimus A9-eluting stents [(Nobori, Terumo Corporation, Tokyo, Japan), and (Biomatrix Flex, and Biomatrix Neoflex, Biosensors Interventional Technologies Pte Ltd., Singapore)]. Nobori, Ultimaster, Biomatrix Flex, Biomatrix Neoflex, and Orsiro were grouped together as biodegradable polymer DES in this study. The DAPT was prescribed before and after the index procedure with lifelong low-dose aspirin (100 mg) in addition to a thienopyridine (75 mg of clopidogrel daily or 90 mg of ticagrelor twice daily) for ideally 12 months but a minimum of 6 months following DES implantation.

The baseline demographic data, clinical outcomes included in-hospital mortality, and long-term mortality, myocardial infarction (MI), target lesion failure (TLF), target lesion revascularization (TLR), target vessel revascularization (TVR) and any revascularization were retrospectively reviewed in detail and collected from medical records in the hospital database. These patients had their regular clinical follow-up every 1 to 3 months according to their conditions at our hospital or returned to the clinics or CV outpatient department of other hospitals where they usually visited before and had regular follow-up. In-hospital outcomes were carefully retrieved by reviewing the medical records and charts, and the clinical outcomes in the post-hospitalized periods were inquired from the notes of outpatient department saved in the hospital database or phone call by the assistant researcher/nurse at study period in case the patients had clinical follow-ups at other hospital. MI is diagnosed by the criteria of universal definition [9] during the follow-up period. TLR is defined as any repeat percutaneous intervention of the target lesion or bypass surgery of the target vessel performed for restenosis or other complication of the target lesion. TVR is defined as any repeat percutaneous intervention or surgical bypass of any segment of the target vessel. TLF is defined as the combination of cardiac death, target vessel MI, or clinically driven TLR. Any revascularization is defined as any repeat percutaneous intervention or bypass surgery for restenosis of the target lesion, or de novo lesion(s) of the target vessel or non-target vessel. Repeated coronary angiography was clinically-driven performed if the patients presented typical angina and suspected a recurrent ischemia event within 1 year or ischemically-driven performed with evidence of coronary ischemia by non-invasive test more than 1 year after the last coronary intervention. (*Reviewer #1, Comment #2 and Comment #5)* The study protocol was reviewed and approved by the Institutional Review Board/Ethics Committee of Taichung Veterans General Hospital, Taichung, Taiwan.

### Statistical analysis

Continuous variables are presented as mean ± standard deviations if normally distributed or median with interquartile range if not. Categorical variables are presented as numbers and percentages. Continuous variables were analyzed by Student-t test or Mann-Whitney test according to data distribution. Categorical variables were analyzed by Chi-square test. Logistic regression analyses was used to determine the independent factors for TVR, TLF, and total mortality in the follow-up period. A *P*-value of less than 0.05 was considered statistically significant. All statistical analyses were performed using SPSS 19.0 (SPSS Inc., Chicago, Illinois, USA) software.

## Results

### Baseline characteristics of patients with Culotte two-stent technique

From July 2008 to November 2015, there were 238 CABD patients undergone culotte two-stent technique in our institute. Of these patients, all DES were implanted for culotte two-stent technique. The baseline characteristics of culotte two-stent patients are shown in Table 1. Most of these patients were elderly, male gender, acute coronary syndrome (ACS) on admission and multiple vessel disease. The characteristics of bifurcation lesions were mostly located at left coronary artery (51.3%), categorized as true bifurcation lesion (92%) and calculated less than 70 degree of bifurcation angle (74.4%).

**Table 1:** Demographic characteristics of coronary artery bifurcation disease patients undergone culotte two-stent technique

	Patients (*N* = 238)
Age, years	70 ± 12
Male, N (%)	183 (76.9)
Diagnosis at admission	
SCAD, N (%)	110 (46.2)
ACS, N (%)	128 (53.8)
CAD vessel numbers	
1-vessel, N (%)	50 (21)
2-vessel, N (%)	100 (42)
3-vessel, N (%)	88 (37)
Multiple vessel, N (%)	188 (79.0)
Location of bifurcation disease	
LM, N (%)	90 (37.8)
LAD-pm, N (%)	94 (39.5)
LAD-md, N (%)	94 (39.5)
LCX-pm, N (%)	3 (1.3)
LCX-md, N (%)	18 (7.6)
RCA-crus, N (%)	5 (2.1)
LM disease, N (%)	90 (37.8)
Bifurcation type	
Medina classification	
1,1,1, N (%)	150 (63)
1,0,1, N (%)	21 (8.8)
0,1,1, N (%)	50 (21)
1,1,0, N (%)	16 (6.7)
0,0,1, N (%)	1 (0.4)
1,0,0, N (%)	0 (0)
True, N (%)	221 (92.8)
Angle < 70 degree, N (%)	177 (74.4)
DK-Culotte, N (%)	87 (36.6)
Stent type.	
DES, N (%)	238 (100%)
Polymer degradable DES, N (%)	41 (17.2)
Hypertension, N (%)	192 (80.7)
Diabetes mellitus, N (%)	111 (46.6)
Smoking, N(%)	116 (48.7)
Hyperlipidemia with Statin therapy, N (%)	156 (65.5)
CKD, N (%)	55 (23.1)
Old CVA, N (%)	36 (15.1)
Old MI, N (%)	50 (21.0)
Prior PCI, N (%)	93 (39.1)
Prior CABG, N (%)	5 (2.1)
BMI, kg/m^2^	24.9 ± 3.5
Serum creatinine, mg/dL	1.2 ± 0.7
Hemoglobin, IU/L	12.6 ± 2.2
LVEF, %	51 ± 11

*SCAD* stable coronary artery diease, *ACS* acute coronary syndrome, *LM* left main, *LAD* left anterior descending, *LCX* left circuflex, *RCA* right coronary artery, *DK* double kissing, *DES* drug-eluting stent, *CKD* chronic kidney disease, *CVA* cerebeler vascular disaese, *MI* myocardial infarction, *PCI* percutaneous coronary intervention, *CABG* coronary arteyr bypass grafting, *BMI* body mass index, *LVEF* left ventricular ejection fraction

### In-hospital and after-discharge clinical outcomes

The observed in-hospital and after-discharge clinical outcomes are shown in Table 2.

**Table 2:** Clinical outcomes of coronary artery bifurcation disease patients undergone culotte two-stent technique

	Patients (N = 238)
In hospital mortality, N (%)	10 (4.2)
Cardiac death, N (%)	6 (60)
Non-cardiac death, N (%)	4 (40)
Total mortality, N (%)	42 (17.6)
Cardiac death, N (%)	10 (23.8)
Non-cardiac death, N (%)	32 (76.2)
Myocardial infarction, N (%)	14 (5.9)
TLF, N (%)	28 (11.8)
TLR, N (%)	27 (11.3)
TVR, N (%)	40 (16.8)
Any revascularization, N (%)	56 (23.5)

*TLF* target lesion failure, *TLR* target lesion revascularization, *TVR* target vessel revascularization

Two hundred twenty-six of two hundred thirty-eight patients (94.9%) completed a medium 3.27 years (mean 3.55 ± 1.94 yr) follow up. The rates of in-hospital mortality and total mortality were 4.2 and 17.6%. Of them, 6 of 10 and 10 of 42 mortalities were caused by cardiac origin causes during the in-hospital and follow-up periods. At post-discharge follow-up, there were 14 (5.9%) cases myocardial infarction, 28 (11.8%) cases TLF, 27 (11.3%) cased TLR, and 40 (16.8%) cases TVR. 56 (23.5%) patients received any revascularization.

### Clinical outcomes of LM bifurcation lesion versus non-LM bifurcation lesion among CABD patients undergone culotte two-stent technique

Among these CABD patients undergone culotte two-stents technique, patients with the LM bifurcation lesion had higher in-hospital and total mortalities than those with the non-LM bifurcation lesion (Table 3). In addition, patients with the LM bifurcation lesion had higher TLF compared to those with the non-LM bifurcation lesion. Prevalence of MI, TLR, TVR or any revascularization was not significantly different between them. *“Reviewer #1, Comment #6”.*

**Table 3:** Clinical outcomes of LM bifurcation lesion versus non-LM bifurcation lesion among coronary artery bifurcation disease patients undergone culotte two-stent technique

	LM lesion *n* = 91	non-LM lesion *n* = 147	*P* value
in hospital mortality, N (%)	8 (8.8)	2 (1.4)	0.015
total mortality, N (%)	29 (31.9)	13 (8.8)	< 0.001
MI, N (%)	6 (6.6)	8 (5.4)	0.607
TLR, N (%)	14 (15.4)	13 (8.8)	0.082
TVR, N (%)	20 (21.9)	20 (13.6)	0.055
TLF, N (%)	15 (16.5)	13 (8.8)	0.048
Any revascularization, N (%)	24 (26.4)	32 (21.8)	0.272

*MI* myocardial infarction, *TLR* target lesion revascularization, *TVR* target vessel revascularization, *TLF* target lesion failure

### Predictors of major adverse cardiovascular and cerebrovascular events

We further analyzed the factors to predict TLF, TVR and total mortality in our cohort study (Tables 4, 5 and 6). For TLF, we found that the angle of bifurcation ≥70° and body mass index (BMI) were positively independent predictors for TLF. For TVR, we found that diabetes mellitus (DM) was an independent predictor to predict TVR in our culotte two-stent patients. For total mortality, we found that statin therapy for hyperlipidemia, hemoglobin and ejection fraction (EF) were negatively independent factors associated to total mortality in our cohort study.

**Table 4:** Logistic regression analysis for clinical outcomes of target lesion failure in all culotte two-stent patients

	Univariate			Multivariate		
	Odds ratio	*P* value	CI	Odds ratio	*P* value	CI
Male gender	0.568	0.200	0.240–1.347	–	–	–
Bifurcation location (LM/non-LM)	2.256	0.046	1.015–5.015	0.797	0.678	0.273–2.324
True/non-true bifurcation	0.634	0.497	0.130–2.362	–	–	–
Bifurcation angle (≥70/< 70)	4.286	< 0.001	1.893–4.795	3.484	0.020	1.213–10.009
DK-culotte	0.760	0.524	0.327–1.767	–	–	–
Admission diagnosis (ACS/SCAD)	2.069	0.090	0.893–4.795	–	–	–
CAD number	1.161	0.585	0.080–1.980	–	–	–
Multiple vessel disease	2.594	0.132	0.749–8.977	–	–	–
LM disease	2.095	0.070	0.940–4.668	–	–	–
DES (absorbed/non-absorbed polymer)	1.128	0.821	0.399–3.186	–	–	–
BMI	1.135	0.028	1.014–1.272	1.136	0.047	1.002–1.289
HT	3.189	0.125	0.726–14.011	–	–	–
DM	3.393	0.006	1.426–8.074	2.219	0.117	0.819–6.008
Hyperlipidemia with statin therapy	0.622	0.249	0.278–1.393	–	–	–
Smoking	0.369	0.024	0.155–0.877	0.517	0.185	0.194–1.373
Old MI	2.132	0.089	0.892–5.095	–	–	–
Old CVA	0.646	0.496	0.184–2.273	–	–	–
Pre-PCI	2.493	0.026	1.116–5.567	1.099	0.845	0.426–2.838
CKD	2.500	0.035	1.065–5.868	0.842	0.789	0.240–2.958
Hemoglobin	0.766	0.004	0.639–0.917	0.825	0.142	0.639–1.066
Serum creatinine	1.335	0.358	0.721–2.472	–	–	–
LVEF	1.012	0.544	0.974–1.051	–	–	–

*SCAD* stable coronary artery diease, *ACS* acute coronary syndrome, *LM* left main, *LAD* left anterior descending, *LCX* left circuflex, *RCA* right coronary artery; *DK* double kissing, *DES* drug-eluting stent, *CKD* chronic kidney disease, *CVA* cerebeler vascular disaese, *MI* myocardial infarction, *PCI* percutaneous coronary intervention, *CABG* coronary arteyr bypass grafting, *BMI* body mass index, *LVEF* left ventricular ejection fraction

**Table 5:** Logistic regression analysis for clinical outcomes of target vessel revascularization in all culotte two-stent patients

	Univariate			Multivariate		
	Odds ratio	*P* value	CI	Odds ratio	*P* value	CI
Male gender	0.619	0.218	0.289–1.327	–	–	–
Bifurcation location (LM/non-LM)	2.000	0.049	1.002–3.990	0.773	0.574	0.315–1.897
True/non-true bifurcation	0.676	0.515	0.208–2.194	–	–	–
Bifurcation angle (≥70/< 70)	2.698	0.007	1.316–5.531	2.303	0.072	0.928–5.714
DK-culotte	0.762	0.463	0.369–1.573	–	–	–
Admission diagnosis (ACS/SCAD)	1.437	0.307	0.717–2.879	–	–	–
CAD number	1.075	0.757	0.681–1.698	–	–	–
Multiple vessel disease	1.756	0.236	0.692–4.457	–	–	–
LM disease	1.750	0.110	0.880–3.479	–	–	–
DES (absorbed/non-absorbed polymer)	1.353	0.495	0.567–3.231	–	–	–
BMI	1.047	0.357	0.950–1.154	–	–	–
HT	1.313	0.571	0.512–3.371	–	–	–
DM	6.333	< 0.001	2.764–14.510	5.358	< 0.001	2.175–13.198
Hyperlipidemia with statin therapy	0.794	0.524	0.390–1.614	–	–	–
Smoking	0.432	0.023	0.210–0.890	0.528	0.124	0.234–1.191
Old MI	2.317	0.031	1.080–4.971	1.499	0.374	0.614–3.660
Old CVA	0.773	0.621	0.280–2.139	–	–	–
Pre-PCI	1.645	0.157	0.826–3.278	–	–	–
CKD	2.232	0.039	1.043–4.779	0.516	0.236	0.173–1.540
Hemoglobin	0.771	0.001	0.658–0.903	0.847	0.125	0.685–1.047
Serum creatinine	1.518	0.113	0.906–2.542	–	–	–
LVEF	0.998	0.892	0.967–1.030	–	–	–

*LM* left main, *DK* double kissing, *ACS* acute coronary syndrome, *SCAD* stable coronary artery diease, *CAD* coronary artery disease, *DES* drug-eluting stent, *BMI* body mass index, *HT* hypertension, *DM* diabetes mellitus, *MI* myocardial infarction, *CVA* cerebeler vascular disaese, *PCI* percutaneous coronary intervention, *CKD* chronic kidney disease, *LVEF* left ventricular ejection fraction

**Table 6:** Logistic regression analysis for clinical outcomes of total mortality in all culotte two-stent patients

	Univariate			Multivariate		
	Odds ratio	*P* value	CI	Odds ratio	*P* value	CI
Male gender	0.629	0.234	0.294–1.349	–	–	–
Bifurcation location (LM/non-LM)	5.448	< 0.001	2.627–11.299	1.412	0.732	0.195–10.219
True/non-true bifurcation	1.697	0.496	0.371–7.769	–	–	–
Bifurcation angle (≥70/< 70)	3.489	0.001	1.725–7.064	0.955	0.937	0.305–2.994
DK-culotte	0.666	0.268	0.325–1.368	–	–	–
Admission diagnosis (ACS/SCAD)	7.259	< 0.001	2.913–18.089	1.727	0.380	0.510–5.848
CAD number	2.339	0.001	1.398–3.914	1.110	0.850	0.376–3.273
Multiple vessel disease	7.121	0.008	1.656–30.622	3.246	0.328	0.307–34.274
LM disease	7.272	< 0.001	3.269–16.180	1.995	0.502	0.266–14.991
DES (absorbed/non-absorbed polymer)	2.464	0.026	1.116–5.438	3.290	0.052	0.997–10.745
BMI	0.894	0.030	0.809–0.989	1.054	0.503	0.904–1.227
HT	0.997	0.994	0.425–2.345	–	–	–
DM	3.239	0.001	1.578–6.647	1.132	0.814	0.403–3.177
Hyperlipidemia with statin therapy	0.352	0.003	0.177–0.701	0.353	0.032	0.136–0.917
Smoking	1.442	0.290	0.732–2.840	–	–	–
Old MI	2.874	0.006	1.363–6.059	0.969	0.958	0.307–3.059
Old CVA	2.855	0.011	1.277–6.382	3.019	0.084	0.862–10.572
Pre-PCI	1.716	0.119	0.830–3.384	–	–	–
CKD	8.639	< 0.001	4.065–18.359	1.467	0.532	0.440–4.891
Hemoglobin	0.578	< 0.001	0.475–0.704	0.658	0.006	0.490–0.885
Serum creatinine	2.856	0.001	1.574–5.184	–	–	–
LVEF	0.924	< 0.001	0.895–0.954	0.938	0.012	0.893–0.986

*LM* left main, *DK* double kissing, *ACS* acute coronary syndrome, *SCAD* stable coronary artery diease, *CAD* coronary artery disease, *DES* drug-eluting stent, *BMI* body mass index, *HT* hypertension, *DM* diabetes mellitus, *MI* myocardial infarction, *CVA* cerebeler vascular disaese, *PCI* percutaneous coronary intervention, *CKD* chronic kidney disease, *LVEF* left ventricular ejection fraction

## Discussion

CABD occurs in 15–20% of CAD patients undergoing PCI remains one of the most challenge complex coronary lesion in interventional cardiology even though there are currently modern interventional stent and technique [1, 2]. One- or two-stent strategy for CABD was currently debated without definite conclusions and two-stent strategy was practically needed for this kind of complex coronary lesion in some specific situations (e.g. severe ostial disease of the side branch, dissection or diminished flow, a large territory at risk in the side branch etc.). The culotte stenting technique as a widely used method was first described in 1998 [10]. Bernard Chevalier et al. used BMS to cover main and side branches with overlapped stents in main branch to treat CABD and had high procedural success rates and good angiographic results with little residual stenosis, but there were high rates of TLF (30%) and TLR (24%) in a short-term follow-up period in their report. In the following decades, there were a few studies report on the culotte technique in the DES era and usually were compared to other two-stent technique for CABD. In these reports, the culotte technique demonstrated lower incidence of angiographic restenosis, lower rates of TLR (2.8%〜14%) and TLF (6.7%〜16.3%), and better clinical outcomes in the DES era [11, 12], and had similar or statistically inferior effects in comparison to other two-stent technique [13–17]. Since there was no definite one strategy superior to other strategy in treating this kind of complex coronary lesion, the culotte two-stent technique (92%) was our major strategy at our institution to treat CABD in specific settings two-stent technique needed, other two-stent techniques (5%) such as T stent, crush, DK-crush, V/Y stent, and simultaneous kissing stent in a minority, and bail-out strategy (2%) for provisional stent strategy but side branch involved in trouble such as reverse crush and T-and-protrusion (TAP) techniques. *“Reviewer #2, Comment #1”* Our experience demonstrated TLF of 11.8%, TLR of 11.3%, in-hospital cardiac death and total death of 6/238 (2.5%) and 10/238 (4.2%) and follow-up period cardiac mortality and total mortality of 10/238 (4.2%) and 42/238 (17.6%) in one arm cohort of a medium 3.27 years follow-up. The TLF, TLR and in-hospital and follow-up period mortalities were relatively high in our study may be mainly contributed to older population, more medical comorbidities, acute setting of CAD, and longer duration of follow-up period resulted in chronological vessel inflammation and had an important impact on all-cause mortality. However, our data also demonstrated the real situations we often face to in the interventional cardiology in a real world.

The culotte technique as one of the most used two-stent technique in specific situations that two stents needed for CABD has several major advantages. First, it allows the operator to start the intervention using a provisional side branch stenting approach and perform bail-out two-stent technique when happening of flow-limited dissection, diminished flow, ostial lesion of side branch non-effective to balloon angioplasty or acute recoil of a side branch during the procedure. Second, the culotte technique provides good protection and stent coverage of a significantly diseased side branch in a large territory at risk when primary two-stent technique were performed. Third, the culotte technique has only two but not three stent layers in the proximal part of the bifurcation lesion, potentially leading to a lower risk of incomplete stent apposition and easier double-rewiring into the side branch with the aim of performing KBT carrying least recoil at the ostium site, least residual stenosis, and less stent distortion in comparison with other techniques. However, there were existed patient characteristics, coronary bifurcation anatomy and stenting techniques contributed to angiographic restenosis and clinical outcomes in terms of MACE and TLR for culotte stenting [12, 18]. Adriaenssens et al. found that older age, increasing bifurcation angle, more severe distal main branch stenosis, and smaller side branch reference diameter were predictors of angiographic restenosis in their culotte stenting cohort [12]. Kawamoto et al. found that bifurcation angle < 70° was not a predictor for MACE in patients with left main coronary bifurcation diseases received mini-crush and culotte stentings [18]. In addition, big bifurcation angle of coronary bifurcation anatomy was an independent factor of follow-up MACE in culotte stenting group compared to other two-stent strategy group in comparing trials designed of different two-stent techniques for CABD. DKCRUSH-III trial demonstrated the 1-year and 3-year MACE rates were significantly higher in the culotte group (6.7 and 14.0%) than that in the DK-crush group (2.4 and 3.8%) among patients with bifurcation angle ≥70° [14]. *“Reviewer #1, Comment #8”* In our culotte stenting experience, we found that bifurcation angle ≥70 degrees and BMI were the independent factors to predict TLF. So increasing/larger bifurcation angle itself was an inherent factor of TLF in culotte technique. Even though we did double kissing technique in the procedure of the culotte stenting, we did not reduce its happening. We postulate that the culotte two-stent technique is technically challenging in these patients with large bifurcation angle with higher rates of stent malapposition and under expansion and are significantly associated with stent stenosis or restenosis. In addition, the abnormal hemodynamic change of shear stress induced by the culotte stenting in those patients with large bifurcation angle might play a central role in the occurrence of TLF.

## Conclusions

The culotte stenting as a major two-stent strategy applied for most LM and left anterior descending artery lesions of our CABD patients is associated with high procedural success, a low relative MACE and acceptable incidence of TLR during an intermediate-term follow up. Big bifurcation angle (≥ 70°) (*Reviewer #1, Comment #1)* is an inherently independent predictor of TLF even though DK technique for the culotte stenting and other two-stent strategy replaced needed to be considered in this situation.

### Study limitations

The present study has several limitations. First, this was an observational, retrospective, and cohort study, and there was no other group of two-stent strategy for comparison. As previously mentioned, culotte stenting technique was the majority for coronary bifurcation lesions at our institution and there would be limited patients with other two-stent strategy and thus the heterogeneity of patient groups would be significantly different in comparison. Secondly, the patient number was relatively too small to have powerful impact for clinical and safety endpoints. In addition, no regular angio follow-up but clinical driven angio follow up may underestimate the real prevalence of TLF/TLR/TVR even though we completed a high percentage (94.9%) of clinical follow-up during an intermediate-term period (medium 3.27 yr. follow-up).

## Data Availability

We are not allowed to share original study data publicly because of the hospital and institution policies, but they are available from the corresponding author on reasonable request.

## Abbreviations

ACS: Acute coronary syndrome
AMI: Acute myocardial infarction
BMI: Body mass index
BMS: Bare metal stent
CABD: Coronary artery bifurcation disease
CAD: Coronary artery disease
DES: Drug-eluting stent
DM: Diabetes mellitus
EF: Ejection fraction
MACE: Major adverse cardiovascular events
PCI: Percutaneous coronary intervention
TLF: Target lesion failure
TLR: Target lesion revascularization
TVR: Target vessel revascularization
